# Magnetic multilayer hydrogel oral microrobots for digestive tract treatment

**DOI:** 10.3389/frobt.2024.1392297

**Published:** 2024-04-12

**Authors:** Ziheng Xu, Zehao Wu, Zichen Xu, Qingsong Xu

**Affiliations:** ^1^ Pui Ching Middle School, Macau, Macao SAR, China; ^2^ Department of Electromechanical Engineering, Faculty of Science and Technology, University of Macau, Avenida da Universidade, Macau, Macao SAR, China

**Keywords:** magnetic microrobot, target delivery, on-demand release, multilayer hydrogel microrobot, digestive tract treatment

## Abstract

Oral administration is a convenient drug delivery method in our daily lives. However, it remains a challenge to achieve precise target delivery and ensure the efficacy of medications in extreme environments within the digestive system with complex environments. This paper proposes an oral multilayer magnetic hydrogel microrobot for targeted delivery and on-demand release driven by a gradient magnetic field. The inner hydrogel shells enclose designated drugs and magnetic microparticles. The outer hydrogel shells enclose the inner hydrogel shells, magnetic microparticles, and pH neutralizers. The drug release procedure is remotely implemented layer-by-layer. When the required gradient magnetic field is applied, the outer hydrogel shells are destroyed to release their inclusions. The enclosed pH neutralizers scour the surrounding environment to avoid damaging drugs by the pH environment. Subsequently, the inner hydrogel shells are destroyed to release the drugs. A set of experiments are conducted to demonstrate the wirelessly controllable target delivery and release in a Petri dish and biological tissues. The results demonstrated attractive advantages of the reported microrobot in microcargo delivery with almost no loss, remote controllable release, and drug protection by the pH neutralizers. It is a promising approach to advance next-generation precision oral therapies in the digestive system.

## 1 Introduction

Oral delivery is the most commonly used route for drug absorption in disease treatments (Jain, 2008; Gupta et al., 2009; Alqahtani et al., 2021). Drug formulations within a human’s complex digestive system will reach various tissues and environments, ranging from the buccal cavity to the intestine and from acid to alkaline environments. However, drugs under standard oral delivery cannot succeed in reaching designated lesions and achieving precise release in the target site. Considering several specific physicochemical properties of drug formulations, strong acid environments will affect the drug’s efficacy or make it useless (Ahadian et al., 2020; Brown et al., 2020; Lang et al., 2020). How to deliver drugs to lesions precisely, achieve on-demand release of them, and protect the drugs in extreme environments is essential to improve the treatment efficiency.

At present, capsule structures are widely utilized in oral drug delivery. It helps to protect the enclosed drug formulations, modify the drug dissolution and stability, and control the release to some extent. There are two steps for the release and absorption of enclosed drug formulations: the disintegration of the capsule’s shell and the drug diffusion. This procedure severely relies on the environmental properties and processing time, which is almost impossible to be precisely controlled. For example, after the patient swallows a capsule, obtaining an accurate predictive model of the capsule disintegration is inaccessible. Thus, researchers have paid great attention to target drug delivery and on-demand release. Since the magnetic actuation has the merits of high penetration, harmless, and high controllability, it is widely applied to navigate drugs toward the target site (Yang and Zhang, 2020; Wang et al., 2021; Wu et al., 2022). After arriving at the target site, the next issue lies in how to release the drugs on demand. Normally, wireless on-demand release can be achieved by different response mechanisms. For the response mechanisms using environmental responses, e.g., biological reaction response (Shigemitsu et al., 2020; Mo et al., 2021) and pH response (Qian et al., 2019; Bhattacharyya et al., 2020; Han et al., 2020), they trigger the drug release by environment factors at the target site. However, similar environments outside the target site may also trigger the drug release. Hence, such release mechanisms are highly dependent on the specificity of the target site. Another type of response mechanism is to control the drug release through external stimuli. The typical response mechanisms are ultrasound response (Arrizabalaga et al., 2022; Yeingst et al., 2022) and light-heat response (Zhao et al., 2020; Zhu et al., 2020). However, they are challenging to achieve controllable release in the deep digestive tract due to the defects of low conductivity of acoustic field and low penetration of light. Recently, our group utilized an alternating magnetic field with a high magnetic gradient to achieve a controllable release of the magnetic hydrogel microrobot in deeper regions (Xu et al., 2023). After the breakage of the capsule’s shell, the drug tends to be affected by external environments within the digestive system, which reduces the therapy efficiency. In the literature, researchers attempted to solve this problem by chemical modifications (Cao et al., 2019; Durán-Lobato et al., 2020) or mechanical injection (Hashim et al., 2019; Lee et al., 2020). However, the effectiveness of chemical modification and the damage caused by mechanical injection remain controversial. Hence, it is desirable to introduce a new method, which imposes no additional chemical modification and mechanical damage. To this end, in this paper, we introduce a novel microrobot with step-by-step release of different inclusions, which enables establishing a suitable local environment around the lesions before releasing the drugs.

**FIGURE 1:**
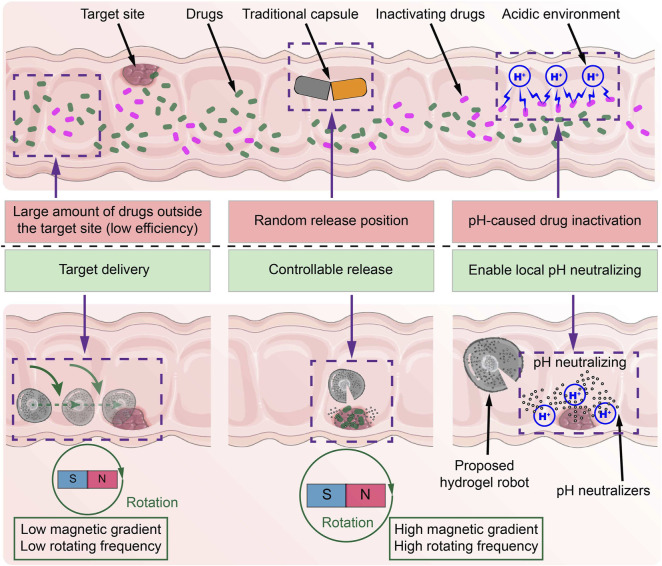
Schematic diagram of the differences between the common capsule and the proposed MMHM in the digestive tract.

The main contribution of this paper is the design of a novel oral multilayer magnetic hydrogel microrobot (MMHM) for digestive tract treatment (see Figure 1). It encloses the pH neutralizers and drugs in the outer and inner hydrogel layers, respectively. By applying different programming magnetic fields, the proposed hydrogel microrobots can be navigated in the digestive tract or disintegrated at the target site. Under an external magnetic field with high rotating frequency and high magnetic gradient, the outer layer can firstly release the pH neutralizers to scour the surrounding environment to avoid damaging the drugs by the pH environment. Subsequently, the inner layer releases its enclosed drugs for precise treatment of diseases. Potential microcargoes include drugs, particles, and other desirable materials. The proposed hydrogel microrobot is safe, easier, and can provide more efficient and precise drug formulation release than the current capsule drug, advancing next-generation target delivery by oral administration.

## 2 Materials and methods

### 2.1 Materials

The magnetic particles were iron oxide (Fe_3_O_4_) particles (diameter: < 0.5 µm). Sodium alginate ((C_6_H_7_NaO_6_)n, 99.5%), sodium carbonate ((Na_2_CO_3_, 99.8%)) and calcium chloride (CaCl_2_, 99.7%) were purchased from Sinopharm Chemical Reagent Co., Ltd. Approval of all ethical and experimental procedures and protocols was granted by the Research Ethics Committee of the University of Macau under Application No. APP-ARE-057 and performed in line with the Animal Protection Act enacted by the Legislative Council of Macao Special Administrative Region under Article 71 (1) of the Basic Law.

### 2.2 Fabrication

Without other specifications, the concentrations of sodium alginate solution and CaCl_2_ solution were 0.375% and 0.25%, respectively; the weight ratio between Fe_3_O_4_ and CaCl_2_ solution (0.25%) was 1: 1.

### 2.3 Simulation study

The software COMSOL 5.6 was utilized for the two-dimensional (2D) computational fluid dynamic simulation studies. To further compare the results of locomotion study, there was a circle placed in a cuboid with a length of 35 mm and a width of 10 mm. The circle was 100 µm close to the bottom. The cuboid was filled with pure water. The boundary conditions of the right, left, and bottom planes of the cuboid were defined as no-slip. The initial state of the pure water was set as static. The solver was determined to find the state of the fluid when it reached steady state.

### 2.4 Magnetism study

A custom-built three-axis Helmholtz coils device (see Supplementary Figure S1, Supplementary Material) was utilized to provide a uniform magnetic field. In addition, the generated maximum magnetic intensity and workspace size are 10 mT and 10 cm cube, respectively. The current of each coil was controlled through six servo amplifiers (model: ESCON 70/10, from Maxon Motor AG.) with signal input from a personal computer (model: OptiPlex 9020, from Dell Technologies Inc.) fitted with a driving board (model: PCIe-6259, from National Instruments Corp.). The velocities were measured by image processing.

### 2.5 Disintegration study

A permanent magnet of a NdFeB cylinder with circular axial magnetization (30 mm in length and 30 mm in diameter) was utilized to provide the magnetic gradient (see Supplementary Figure S2, Supplementary Material). In addition, a brushless motor (model: 57BL06Y55-230D, from Hai Chuang Jia Jie (Beijing) Technology Co., Ltd.) was utilized to rotate the permanent magnet with the rotatory axis perpendicular to the circular axis. The magnetic gradient was changed through increasing or decreasing the distance between the microrobot and the rotation axis of the permanent magnet. In addition, the magnetic gradient was derived by the measured magnetic flux density from a 3D magnetic sensor (model: TLV493D-A1B6, Infineon Technologies AG). The given magnetic gradients in this study are the mean values of one rotation cycle (see Supplementary Figure S3, Supplementary Material).

### 2.6 Drug protection study

Calcium hydroxide (Ca(OH)_2_, 95%, Tianjin Bodi Chemical Co., Ltd.) was utilized to create alkaline environments. Hydrochloric acid (HCl, 1 M, Yida Technology (Quanzhou) Co., Ltd) was utilized to create acid environments. A constant-temperature heating device (DXY-1515, Shenzhen Dingxinyi Experimental Equipment Co., Ltd) was used to create 40°C environments.

### 2.7 pH-neutralizing study

The concentration of CaCl_2_ solution to the suspension of the outer hydrogel shell was 0.5%, then Na_2_CO_3_ was ultrasonically mixed into the CaCl_2_ solution with the concentration of 0.25% to generate highly dispersed calcium carbonate (CaCO_3_) powders. 7-hydroxy coumarin (5.0 µM) was mixed into the CaCl_2_ solution to the suspension of the inner hydrogel shell.

### 2.8 Data analysis and statistics

Unless otherwise specified, all experiments were repeated at least three times independently.

## 3 Results

### 3.1 Fabrication of the multilayer hydrogel microrobot

**FIGURE 2:**
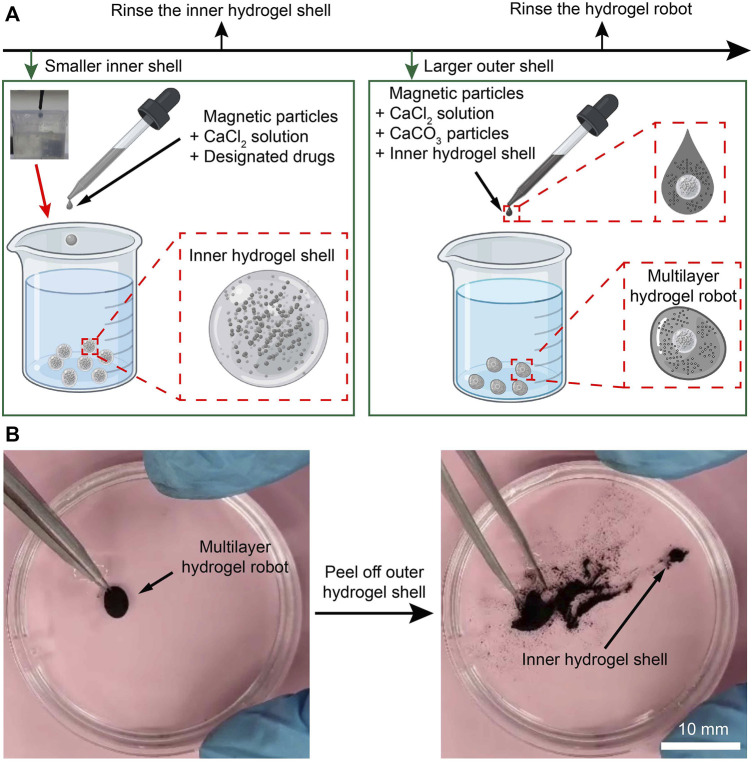
Fabrication of the MMHM. **(A)** Schematic diagram of the fabrication procedure. **(B)** Prototype of the multilayer hydrogel microrobot.

To deliver drugs to lesions and protect and release them precisely, the concept design of a multilayer microrobot is proposed by containing the pH neutralizers and the relatively small magnetic hydrogel microrobots (with drugs enclosed) in a larger hydrogel shell. The fabrication procedure of the multilayer microrobot is shown in Figure 2A.

First, the CaCl_2_ solution, drugs, and Fe_3_O_4_ magnetic microparticles were added together to produce a suspension. Then, the suspension droplet was added to the sodium alginate solution to generate the inner hydrogel shell (Figure 2A, inserted picture in the upper-left corner). Pure water was used to rinse the inner hydrogel shell. These microrobots are relatively smaller than the outer hydrogel shells so that the outer hydrogel shells can enclose them. Afterward, a suspension with the inner hydrogel microrobot, CaCl_2_ solution, pH neutralizers (CaCO_3_), and magnetic microparticles were added to the sodium alginate solution again to generate the outer hydrogel shell, namely, the relatively larger magnetic hydrogel microrobots. Rinsed by pure water again, a MMHM was obtained. Notably, the CaCl_2_ solution and sodium alginate solution are biocompatible. In addition, the Fe_3_O_4_ microparticles are biocompatible, innocuous, and biodegradable.

To demonstrate the success of enclosing the inner hydrogel shell, the outer hydrogel shell was peeled off to show its inclusions. As shown in Figure 2B, the inner hydrogel shell was revealed, verifying the effectiveness of the fabrication procedure. The verification procedure is demonstrated in Supplementary Material S1, Supplementary Material.

### 3.2 Characterization of toxicity and magnetism property

**FIGURE 3:**
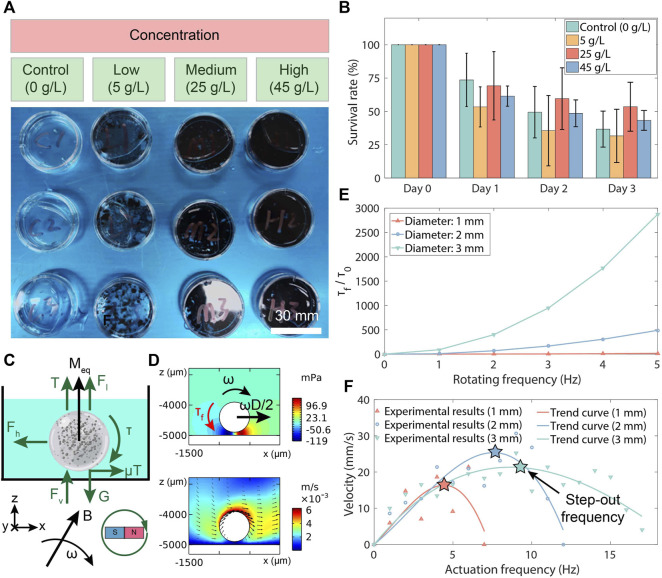
Toxicity and magnetism test results of MMHM. **(A)** Experimental setups of the toxicity test. **(B)** The survival rates of the co-cultured zebrafish embryos at various co-cultured concentrations of disintegrated MMHMs. **(C)** Force analysis of an MMHM rolled in a fluid. **(D)** Simulation results of the pressure and flow rate distributions around the hydrogel microrobots. **(E)** The 2D fluid-based resistance torque (*τ*_*f*_, torque per unit depth) for the hydrogel microrobots with various diameters *versus* rotating frequency. *τ*_*f*_ is normalized with respect to a constant *τ*_0_ = 6.27 × 10^−8^ N, i.e., resistance torque of the microrobot with a diameter of 1 mm at a rotating frequency of 1 Hz. **(F)** Relationship of velocity of hydrogel microrobots with different diameters *versus* the rotating frequency.

To examine the toxicity of the fabricated MMHM, different concentrations of hydrogel microrobots were disintegrated and co-cultured with zebrafish embryos in pure water (Figure 3A). By observing and recording the survival number of the embryos, the biocompatibility of the proposed hydrogel-based microrobot design is evaluated. The survival rate was calculated as the percentage of the survived over total numbers of zebrafish embryos. Three groups of experiments were conducted with the microrobot suspensions at different concentrations. The suspension was produced by pounding microrobots into pieces within the water. The results indicate that the generated microrobot is biocompatible without toxicity because the survival result exhibits no significant reduction with increasing co-cultured concentrations of hydrogel microrobots (Figure 3B).

For precision delivery, the microrobots are driven to roll on the digestive tract surface by a rotating uniform magnetic field produced by a custom-built three-axis Helmholtz coils device. The hardware connection scheme is shown in Supplementary Figure S4, Supplementary Material. The force analysis procedure of the microrobot is shown in Figure 3C. The buoyancy and gravity are presented as *F*_*l*_ and *G*, respectively. According to Bernoulli’s principle, the fluidic pressure is decrease as the nearby flow rate increases. Thus, with the flow rate distributions in this case (see Figure 3D), there is a vertical flow force (*F*_*v*_) and a horizontal flow force (*F*_*h*_), which can be expressed as:

$$\left(F_{v},F_{h}\right)\propto \left(P_{v},S\right)\propto \omega^{2}D^{4}$$ (1)

where *P*_*v*_ and *S* are the equivalent vertical pressure difference and surface area of the microrobot, respectively. *ω* and *D* are the rotation frequency and diameter of the microrobot, respectively. In addition, *F*_*h*_ represents as the drag force obtained from the fluidic environment. Then, the supporting force of the bottom to the hydrogel microrobot (*T*) can be derived as:

$$T=G-F_{l}-F_{v}=\left(\rho_{r}-\rho_{f}\right)Vg-F_{v}$$ (2)

where *ρ*_*r*_ and *ρ*_*f*_ are the densities of the hydrogel microrobot and the fluid, respectively. Besides,*V* is the volume of the hydrogel microrobot, and *g* is the gravity coefficient.

The equivalent friction coefficient is assigned as *μ*. Thus, the friction can be denoted as *μT*. Assume that the rotation frequency is equal to the step-out frequency of the microrobot, the torques can be expressed as follows.

$$\left\{\begin{array}{cc} & \tau =\tau_{f}\\ & \tau_{f}=\frac{1}{2}F_{h}D=\frac{1}{2}\mu TD\\ & \tau =\left(m_{o}V\right)\times B=\left(\frac{1}{6}m_{o}\pi D^{3}\right)\times B \end{array}\right.$$ (3)

where *τ* and *τ*_*f*_ are the magnetic torque and flow-resistant torque, respectively. **m**_**o**_ is the equivalent magnetization of the hydrogel microrobot. **B** is the applied magnetic field.

The simulation results of the pressure and flow rate distributions of a rotating hydrogel microrobot are depicted in Figure 3D. Based on the simulation results, the tendencies of *τ*_*f*_ with various diameters of the microrobot and rotating frequencies of the magnet are shown in Figure 3E. It is observed that the flow-resistant torque increases as the microrobot diameter rises, which is consistent with the theoretical prediction by Eqs 1–3. Therefore, the magnetic actuation ability of the hydrogel microrobot is characterized by its step-out frequency.

To demonstrate the influence of the actuation frequency of rotating magnetic field on the velocity of the hydrogel microrobot, the microrobots with different diameters in a Petri dish (filled with pure water) are driven by the rotating magnetic field with a magnitude of 10 mT and various actuation frequencies. The velocities at different actuation frequencies are measured, as shown in Figure 3F. It is seen that the step-out frequency of the hydrogel microrobot increases with the microrobot diameter rising. Therefore, considering the theoretical models in Eqs 1–3, it is found that with the increase in microrobot diameter, the growth of magnetic torque (*τ* ∝ *D* ^3^) is larger than the resistant flow torque (*τ*_*f*_ ∝ *TD*, *T* ∝ (*V*, − *F*_*v*_), *V* ∝ *D* ^3^, *F*_*v*_ ∝ *D* ^4^). In addition, we can obtain a faster rolling speed by applying a larger magnetic intensity. Moreover, it is observed that there are large fluctuations in these velocity-frequency curves. This may result from the continuous shape change of the hydrogel shell due to the magnetic field actuation in the experimental test.

### 3.3 Results of on-demand release by a gradient magnetic field

The hydrogel microrobot can be regarded as a soft hydrogel shell enclosing magnetic fluids inside. In this work, gradient magnetic fields are utilized to actuate the magnetic microrobots, which apply the induced forces to break the hydrogel membranes. The more magnetic microparticles are magnetized, the stronger forces will be applied to the hydrogel membrane by the same gradient magnetic field.

According to the positions of the microparticles relative to the hydrogel membrane, the magnetic microparticles can be divided into two types: microparticles connected with hydrogel structures and microparticles inside the internal fluid (see Supplementary Figure S5, Supplementary Material). When the magnetic fields actuate the microparticles associated with the hydrogel shell, their movement will contribute to the aperture of the hydrogel shell and destroy it. For the microparticles inside the internal fluid, the disintegration procedure can be regarded as the microparticles applying stress on the hydrogel shell and destroying it. The above two procedures contribute to the physical disintegration of the hydrogel microrobots.

**FIGURE 4:**
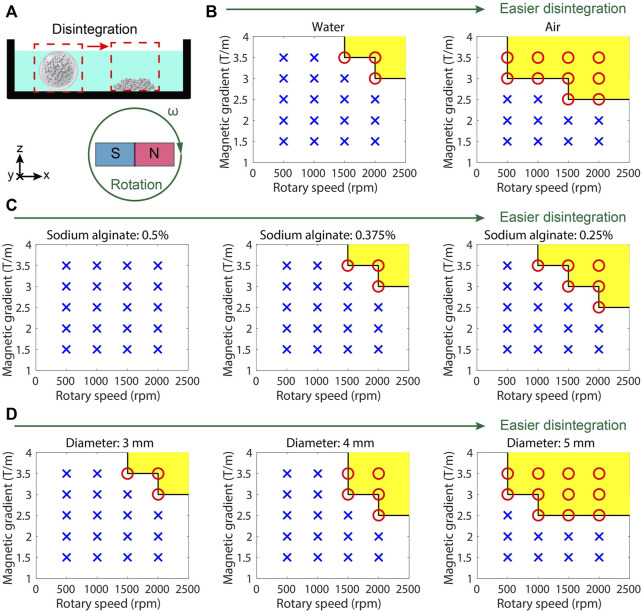
Controllable release test results of MMHM. **(A)** Schematic diagram of the experimental setup. **(B)** Succeeded or unsucceeded disintegrations in different environments under the rotating gradient magnetic field with different frequencies and intensities. **(C)** Succeeded or unsucceeded disintegrations at various concentrations of sodium alginate under the rotating gradient magnetic field with different frequencies and intensities. **(D)** Succeeded or unsucceeded disintegrations of the microrobots with different diameters (size) under different rotating gradient magnetic fields with different frequencies and intensities. Red circle and yellow area: succeeded disintegration. Blue cross: unsucceeded disintegration.

To demonstrate the influences of the magnitude and rotating frequency of the gradient magnetic field on the disintegration of the hydrogel microrobot, the microrobots at different conditions are placed in the rotating gradient magnetic fields with various frequencies and intensities (Figure 4A). The succeeded or unsucceeded disintegrations under different conditions are illustrated in Figures 4B–D. It is found that in a dry environment, the hydrogel microrobot is much easier to disintegrate (Figure 4B) because there is no buoyancy to keep its shape stable, and the friction at the bottom increases. With the increase in the concentration of sodium alginate, the hydrogel microrobot becomes difficult to disintegrate (Figure 4C), because a high concentration of sodium alginate will increase the reaction intensity to enhance the strength of the hydrogel shell. Moreover, the hydrogel microrobot with a larger size (Figure 4D) is easy to disintegrate because the magnetic force is enhanced with the increasing level of Fe_3_O_4_ microparticles. It proves that the outer hydrogel shell will be disintegrated before the inner hydrogel shell under a programming magnetic field.

### 3.4 Results of drug protection in extreme digestive environments

**FIGURE 5:**
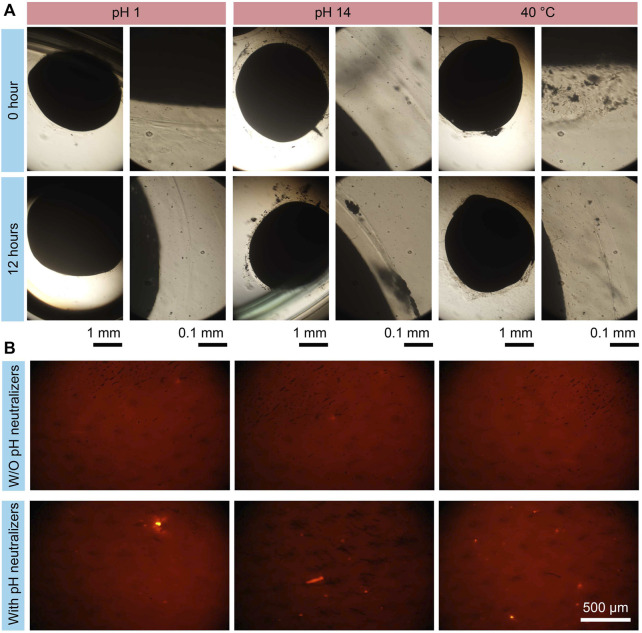
Drug protection test results of MMHM in the extreme environments. **(A)** Structure stability test result of hydrogel microrobot in an acidic environment, an alkaline environment, and a 40°C environment. **(B)** Fluorescent images of the pH-sensitivity fluorescent probes in a pH = 4 environment under the on-demand releasing with or without carrying the pH neutralizers.

To demonstrate the stability of the proposed hydrogel microrobots in extreme digestive environments, the microrobots were placed in an acid environment (e.g., in the stomach), an alkaline environment (e.g., in the pancreatic duct), and a 40°C environment (e.g., at high fever), as detailed in Figure 5A. Acidic fluid environments were formed using the HCl solution, and alkaline environments were created by adopting the Ca(OH)_2_ solution. The hydrogel structures stayed stable in pH = 1, pH = 14, and 40°C environments for more than 12 h. These results demonstrate the stability of the proposed microrobots, enabling further applications in the digestive system.

To reveal the effectiveness of the pH neutralizers carried by the microrobots, the inner hydrogel shells enclose the fluorescent probes with pH sensitivity (7-hydroxy coumarin). The multilayer microrobots with or without pH neutralizers were released in a pH = 4 environment. The fluorescent images of the fluorescent probes are shown in Figure 5B. It is observed that the fluorescent signals without carrying pH neutralizers are much weaker than that with pH neutralizers. The results indicate that the proposed design concept of multilayer microrobot for carrying pH neutralizers can effectively prevent drug damage due to the pH environment.

### 3.5 Targeted delivery and on-demand release in a biological environment *in Vitro*

**FIGURE 6:**
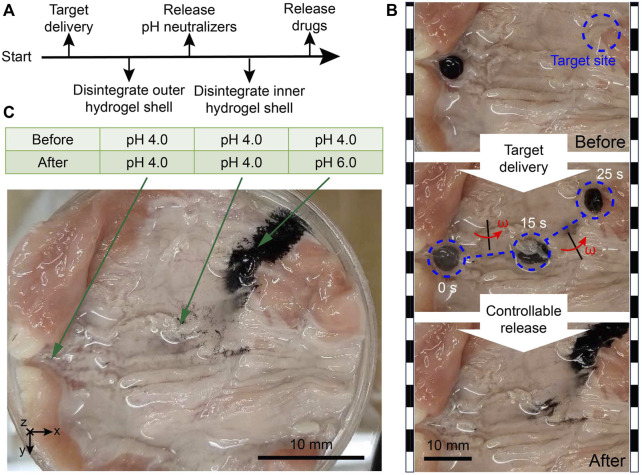
Biological tissue environmental test results of MMHM. **(A)** Schematic diagram of the operation procedure. **(B)** Snapshots of the experimental test. **(C)** pH measurements in the cow’s intestine before and after the local environmental adjustment.

In the digestive system, the irregular tissue morphology and accompanying secreted mucus are unavoidable, which can lead to severe microcargo loss during the delivery process. For illustration, the microrobot-based target delivery and controlled release tasks have been conducted in a cow’s intestine *in vitro*, as illustrated in Figure 6 and Supplementary Material S1, Supplementary Material. It is evident that under the protection of hydrogel shells, there is almost no microparticle loss during the navigation procedure. External gradient magnetic fields can release nearly all the enclosed materials at the designated position. Moreover, the magnitudes of pH values at three places in the cow’s intestine (including the target site) were measured. It is found that the carried outer pH neutralizers (CaCO_3_ particles) had done an expected targeted environmental modification, which can avoid the deactivation of carried inner drugs in acidic environments. In the target area, the proposed design succeeded in creating a relatively mild environment for drugs, contributing to better utilization of medications in extreme environments. In addition, since the pH values of the places (rather than the target site) had no change after the experimental test, we can determine that there was no unexpected release outside the target site.

## 4 Discussion and conclusion

In this paper, a multilayer hydrogel shell-based magnetic oral microrobot is designed to achieve targeted delivery and remotely controlled release when applying proper external gradient magnetic fields. The release procedure can be remotely accomplished step by step. The outer layer structure helps to release smaller microrobots and pH neutralizers so that the environments can be modified first and create a mild condition for drugs. After that, the inner layer structure contributes to releasing the designated drugs. Multilayer microrobots solved the problem that microparticles tend to be stuck to the digestive system’s tissues and mucus. The proposed multilayer microrobots demonstrate attractive cargo delivery and controllable release prospects. It also enables plenty of easily inactivated drugs to make effects in extreme environments. With the help of gradient magnetic fields, the microrobot can reach almost anywhere in the digestive system and release the enclosed drug formulations to use the drug fully. In addition, the enclosed cargoes can be diverse, where it is possible to deliver designated treatment materials and protect them from the environmental changes in the gastrointestinal tract and some ducts of the digestive glands.

At this stage, experimental investigations of the MMHM performed *in vitro* have demonstrated the promising capabilities of targeted cargo delivery and on-demand release. However, the *in vivo* application will encounter more situations. In the future, the influence of other foods inside the digestive system on the microrobots under different osmotic pressures will be studied. In reality, the digestive tract experiences peristalsis. Such motion will be exerted on the microrobots, and its impact on the integrity of microrobots will be discovered. Moreover, it is hoped to integrate more functional materials in the microrobots to achieve more biomedical applications, such as bio-sensing, diagnosis, and medical imaging, inspiring novel solutions for next-generation therapies in the digestive system.

## Data Availability

The original contributions presented in the study are included in the article/Supplementary Material, further inquiries can be directed to the corresponding author.
