# Supplementary material for: Magnetic multilayer hydrogel oral microrobots for digestive tract treatment
Source: Front Robot AI. 2024 Apr 12;11:1392297. doi: 10.3389/frobt.2024.1392297 (PMC11045901; doi:10.3389/frobt.2024.1392297)
Supplement: Supplementary file 3 [file Image1.pdf]

# Supplementary Material

## 1 SUPPLEMENTARY FIGURES

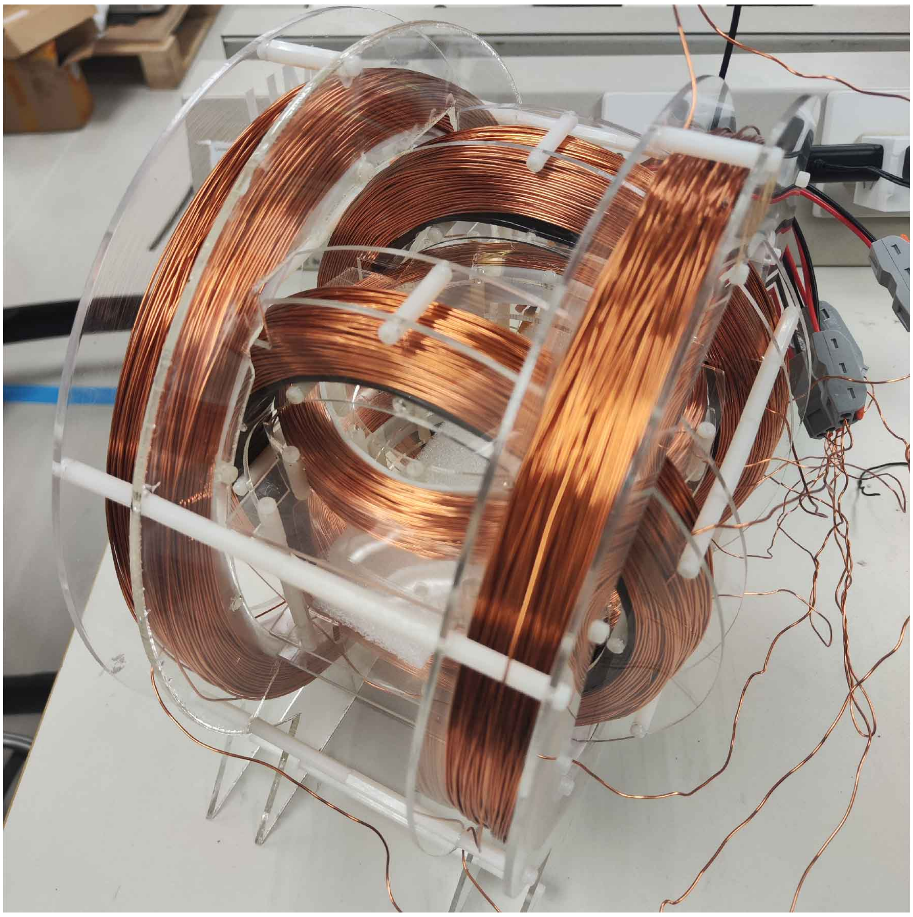

**Figure S1.** The custom-built three-axis Helmholtz coils device.

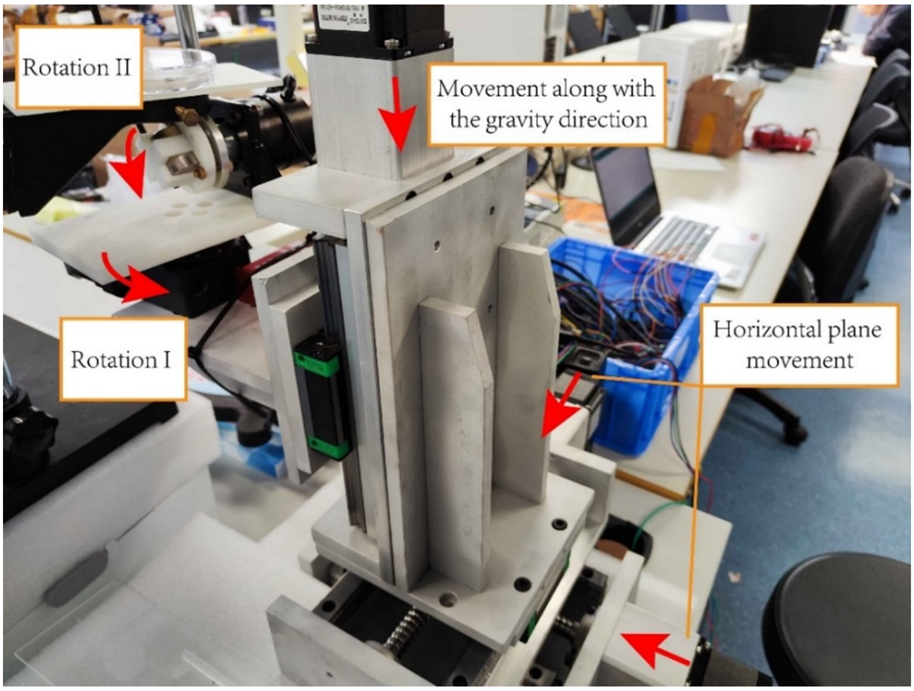


**Figure S2.** Experimental setup of the gradient magnetic field actuation system.

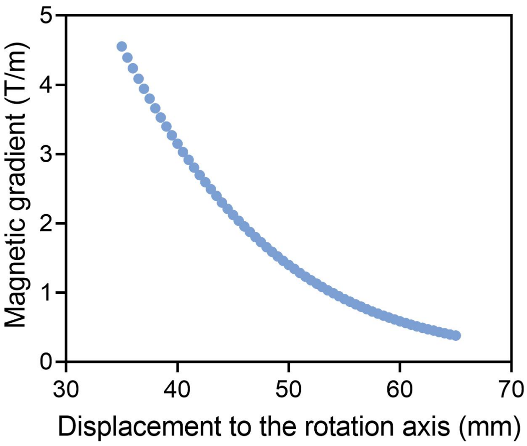


**Figure S3.** Relationship between the magnetic gradient value and the displacement to rotation axis of the permanent magnet.

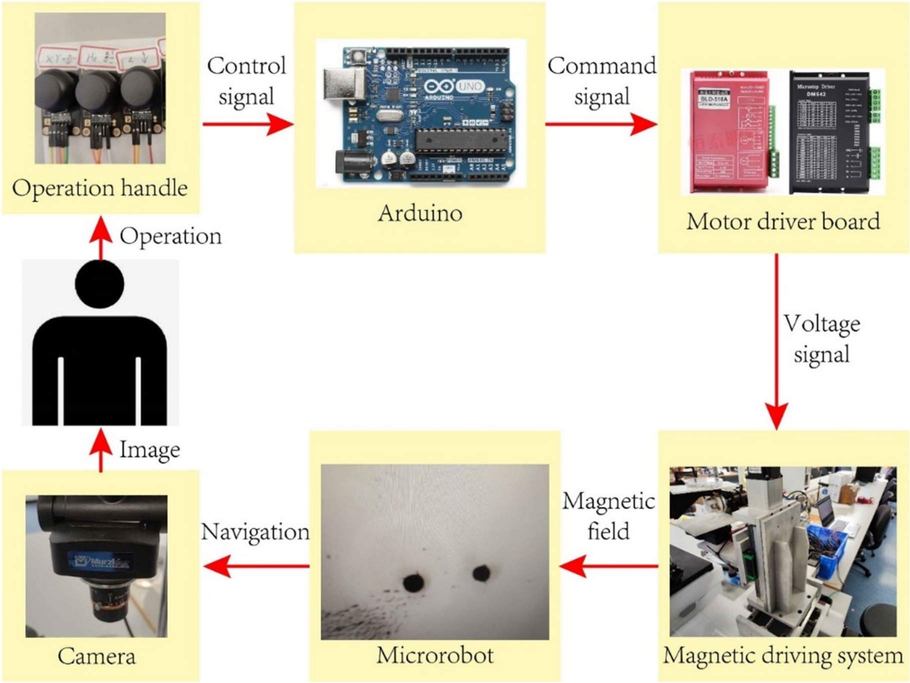


**Figure S4.** Connection scheme of the fabricated magnetic actuation system.

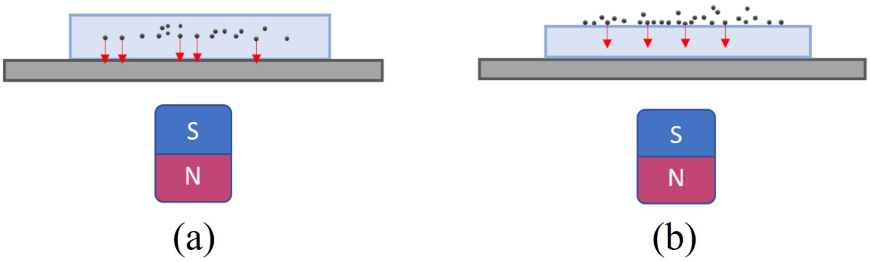


**Figure S5.** Illustration of the disintegration of hydrogel shells by gradient magnetic field.
